# LPM682000012, a Synthetic Neuroactive Steroid That Ameliorates Epileptic Seizures by Downregulating the *Serpina3n*/NF-κB Signaling Pathway

**DOI:** 10.3390/molecules29225286

**Published:** 2024-11-08

**Authors:** Xiaofan Zhang, Shengmin Ji, Yue Yang, Xiaohui Sun, Hui Wang, Yifan Yang, Xuan Deng, Yunjie Wang, Chunmei Li, Jingwei Tian

**Affiliations:** 1 Key Laboratory of Molecular Pharmacology and Drug Evaluation, School of Pharmacy, Ministry of Education, Collaborative Innovation Center of Advanced Drug Delivery System and Biotech Drugs in Universities of Shandong, Yantai University, Yantai 264005, China; zhangxfan2022@163.com (X.Z.); jsm7777@126.com (S.J.); ytuyy1203@163.com (Y.Y.); sxh163yx2022@163.com (X.S.); yangyifan23119@163.com (Y.Y.); dx19811718896@163.com (X.D.); yjwcpu@163.com (Y.W.); 2 R & D Center, Luye Pharma Group Ltd., Yantai 264003, China; wanghuisdyt@126.com

**Keywords:** epilepsy, PAM, GABA_A_ receptor, NASs, *Serpina3n*, NF-κB

## Abstract

Epilepsy is characterized by abnormal neuronal firing in the brain. Several therapeutic strategies exist for epilepsy; however, several patients remain poorly treated. Therefore, the development of effective treatments remains a high priority in the field. Neuroactive steroids can potentiate extra-synaptic and synaptic GABA_A_ receptors, thereby providing therapeutic benefits relative to benzodiazepines. This research study investigated the therapeutic effectiveness and underlying mechanisms of LPM682000012, a new synthetic neuroactive steroid-positive allosteric modulator (PAM) of GABA_A_ receptors employed for treating epilepsy. Acute and chronic rat epilepsy models were established to identify the anti-seizure potency of LPM682000012. The dose-dependent sedative effects of LPM682000012 and Ganaxolone in normal rats were evaluated, which revealed that they both dose-dependently alleviated acute epileptic seizure in the pentylenetetrazol (PTZ)-mediated seizure model. Furthermore, LPM682000012 indicated an enhanced safety profile than Ganaxolone. Moreover, LPM682000012 also indicated therapeutic effects in the kainic acid (KA)-induced chronic spontaneous seizure model. Morphologically, LPM682000012 decreased neuronal loss in the hippocampal CA1 and CA3 regions and increased dendritic spine density in the CA1 region. In addition, mechanical analyses, including transcriptomics, Western blot, and proteomics analyses, revealed that the *Serpina3n*/NF-κB signaling pathway was up-regulated in epileptic rat hippocampal tissue, and LPM682000012 treatment reversed these changes. In summary, this report demonstrated that the novel neurosteroid GABA_A_ PAM LPM682000012 activated the synaptic and extra-synaptic GABA_A_ receptors and alleviated KA-induced neuronal loss and synaptic remodeling, potentially by down-regulating the *Serpina3n*/NF-κB signaling pathways. The results provide evidence that LPM682000012 is a potential anti-seizure pharmacotherapy candidate for epilepsy and warrants further research.

## 1. Introduction

Epilepsy is a highly prevalent and serious brain disorder, affecting about 1% of individuals globally. It is defined as recurrent episodic seizures due to aberrant neuronal discharge caused by brain inhibition and excitation imbalance [1]. Currently, pharmacotherapies are the primary treatment choice. Although there are several available anti-seizure drugs (ASDs) on the market, about 30% of patients remain drug-resistant, whereas in temporal lobe epilepsy patients, the drug-resistant rate is >70% [2]. Epileptogenesis is a chronic process that can be triggered by genetic or acquired factors and that can continue long after epilepsy diagnosis [3]. The current ASDs only target seizures and not epileptogenesis, which have undesirable effects [4], resulting in an increased burden on patients and society. Therefore, novel and effective antiepilepsy pharmacotherapies are urgently required for clinical application.

Neuroactive steroids (NASs) affect the central nervous system (CNS) functions via different mechanisms, such as positive allosteric regulation of the GABA_A_ receptor [5,6,7]. The GABA_A_ receptor family is complex, comprising 19 known subtypes and widely distributed in the brain. GABA_A_ receptors are pentameric ion channels primarily comprising three β subunits (β_1_–β_3_), six α (α_1_–α_6_), and additional subunits (γ_1_–γ_3_, δ, ε, π, or θ) [8]. The receptor’s pharmacological and biophysical features, as well as location at synaptic or extra-synaptic sites, depend on the unique subunit composition. Moreover, due to their essential modulatory role in neuronal circuits, these receptors are targeted by various clinically relevant drugs, including anesthetics, barbiturates, and benzodiazepines. The NASs modulate synaptic and extra-synaptic GABA_A_ receptors as well as enhance extra-synaptic GABA_A_ receptor levels via multiple pathways relative to benzodiazepines, which can only target GABA_A_ receptors containing the γ subunit [9]. This multimodal feature of NAS GABA_A_ receptor-positive allosteric modulators (PAMs) provides them with a therapeutic advantage over benzodiazepines.

Because of this differentiation, GABA_A_ receptor-modulating NASs have become a research hotspot profile [10,11,12]. Brexanolone is an endogenous first-generation NAS, which was the first drug authorized by the FDA in 2019 for treating postpartum depression (PPD) [13]. Furthermore, in 2023, the FDA approved Zuranolone (SAGE-217) as the first oral drug to treat PPD patients. Moreover, Ganaxolone is a synthetic allopregnanolone analog [14], which was first authorized in March 2022 in the USA for treating seizures associated with cyclin-dependent kinase-like 5 (CDKL5) deficiency disorder (CDD) in patients aged 2 years and older [15]. 

*Serpina3n*, a murine orthologue of human α-1-antichymotrypsin, is a member of the serpin superfamily of protease inhibitors [1]. *Serpina3n* is recognized as a key component of the inflammatory response in the brain [2]. Recently, *Serpina3n* has been found to be significantly upregulated in neurological diseases, such as epilepsy, traumatic brain injury, and hypothalamus inflammation, and plays an important role in the development of the disease [3,4,5,6]. In epileptic mice after NF-κB inhibitor treatment or pretreatment with *Serpina3n* knockdown, the expression levels of proinflammatory cytokines were decreased [5]. These results indicate that the *Serpina3n*/NF-κB signaling pathway may be associated with epilepsy.

**Figure 1:**
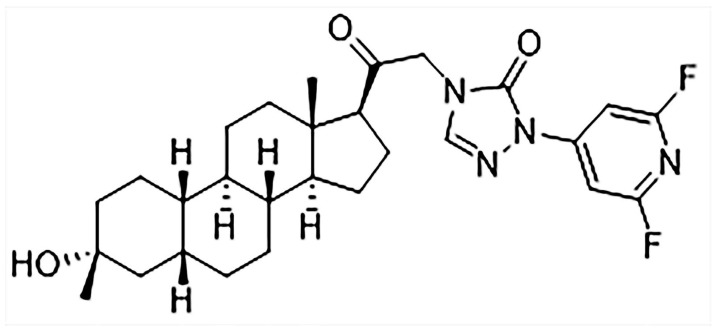
Chemical structure of LPM682000012.

LPM682000012 is a new efficient NAS and an extra-synaptic and synaptic GABA_A_ receptor PAM. It is a difluoropyridyl analog based on the chemical modification and structure–activity relationship (SAR) analysis of SAGE-217 (Figure 1), which increase the molecular stability by adding the adjacent substitution of a pyridine nitrogen atom [16]. LPM682000012 has been observed to have enhanced efficacy (E_max_ = 1067%) and potency (EC_50_ = 75 nM) compared to Ganaxolone (EC_50_ = 94 nM, E_max_ = 225%) on extra-synaptic GABA_A_ receptor activity. Moreover, it has higher synaptic GABA_A_ receptor efficacy (E_max_ = 675%) and potency (EC_50_ = 141 nM) than Ganaxolone (EC_50_ = 256 nM, E_max_ = 307%) [16,17].

This report investigated LPM682000012’s anti-seizure potential against chronic and acute epilepsy models and the associated neurobiological mechanisms. The acquired data provide evidence for the application of LPM682000012 as a novel anti-seizure drug.

## 2. Results

### 2.1. LPM682000012 Produced a Sedative Effect in Rats

**Figure 2:**
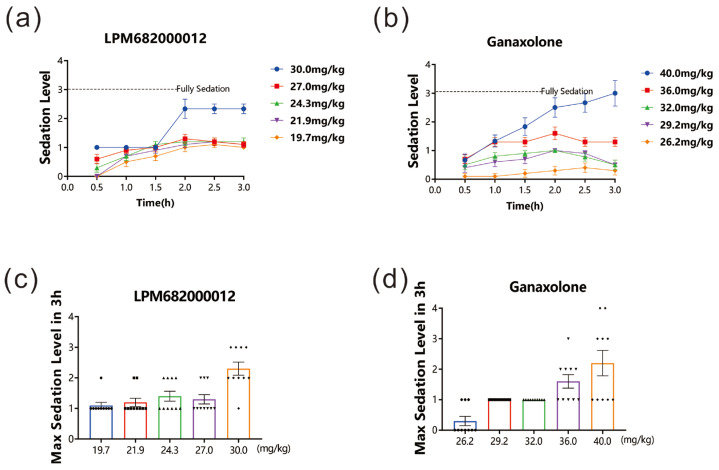
LPM682000012 produced a sedative effect in rats. LPM682000012 or Ganaxolone was administered via i.g., and rats were monitored for sedation for 3 h. (**a**) Sedation levels in LPM682000012-treated and (**b**) Ganaxolone-treated groups. (**c**) Max. sedation level in LPM682000012-treated groups. ● means 19.7 mg/kg, ■ means 21.9 mg/kg, ▲ means 24.3 mg/kg, ▼ means 27.0 mg/kg and ◆ means 30.0 mg/kg. (**d**) Max. sedation level in Ganaxolone-treated groups. ● means 26.2 mg/kg, ■ means 29.2 mg/kg, ▲ means 32.0 mg/kg, ▼ means 36.0 mg/kg and ◆ means 40.0 mg/kg. Results are expressed as mean ± SEM, n = 10.

After drug administration, the sedative effect in rats was evaluated for 3 h. The data revealed that both LPM682000012 (19.7 to 30 mg/kg) and Ganaxolone (26.2 to 40 mg/kg) dose-dependently induced sedative effects. Furthermore, the LORR and unrecoverable within 1 min indicated that 30 mg/kg LPM682000012 and 40 mg/kg Ganaxolone indicated significant sedation 1.5–2 h post-dosing. The sedative effect lasted beyond the 3 h study period (Figure 2a–d).

### 2.2. LPM682000012 Reduced Acute Epileptic Seizure in PTZ Model

**Figure 3:**
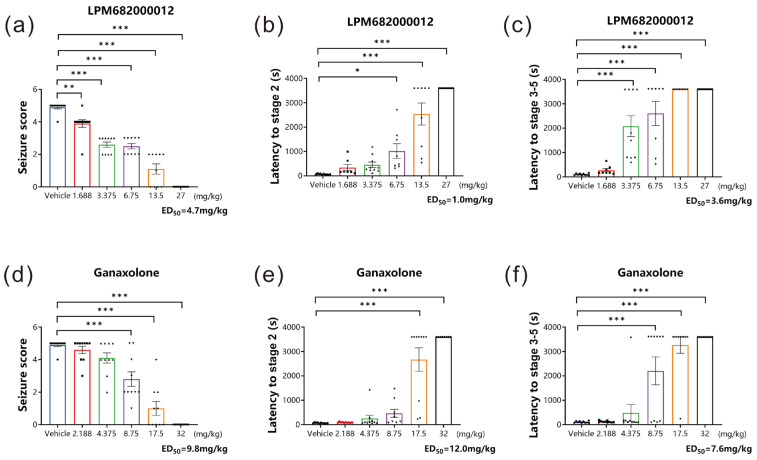
LPM682000012 alleviated acute epileptic seizure in the PTZ model dose-dependently. (**a**,**d**) Seizure scores of LPM682000012 and Ganaxolone. (**b**,**e**) The latency of stage 2 of LPM682000012 and Ganaxolone. (**c**,**f**) The latency of stage 3–5 of LPM682000012 and Ganaxolone. Results are expressed as mean ± SEM, n = 8. * *p* < 0.05, ** *p* < 0.01, and *** *p* < 0.001 vs. Vehicle group. ● means Vehicle group and other symbols represent different doses of the administration group, respectively.

The classical rodent model for preclinical anti-seizure drug screening is established using a GABA_A_ receptor inhibitor, PTZ, which can induce acute generalized myoclonic seizure by reducing the inhibitory synaptic transmission to increase neural excitability [18]. In this research study, the PTZ-induced acute epileptic seizure model was established to assess if LPM682000012 had in vivo anticonvulsant activity. Based on sedative analysis results, non-sedative doses of LPM682000012 and Ganaxolone were analyzed. It was found that LPM682000012 dose-dependently reduced the seizure score (*p* < 0.001) and extended the stage 2 (*p* < 0.001) and the stage 3–5 latency (*p* < 0.001) during the 1 h observation period (Figure 3a–c). Ganaxolone also showed similar results (Figure 3d–f, *p* < 0.001).

The ED_50_ values of LPM682000012 for seizure scores, the latency of stage 2, and the latency of stages 3–5 were 4.7, 1.0, and 3.6 mg/kg, respectively, whereas for Ganaxolone, the ED_50_ values were 9.8, 12.0, and 7.6 mg/kg, respectively. The LPM682000012 and Ganaxolone safety margins were calculated based on doses that do not produce loss of consciousness in rats divided by the anti-seizure ED_50_ values (Table 1).

### 2.3. LPM682000012 Attenuated KA-Induced Chronic Spontaneous Seizures in Rats

**Figure 4:**
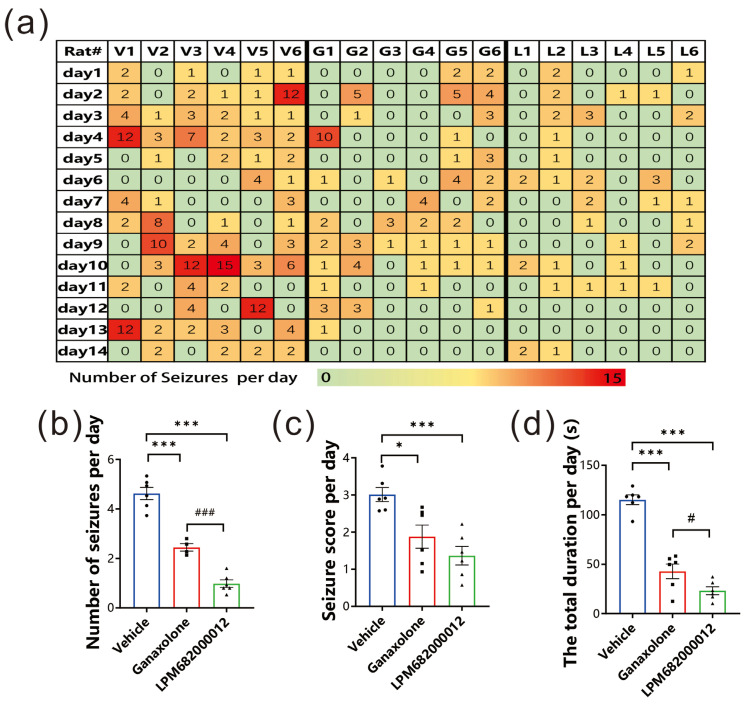
LPM682000012 attenuated spontaneous seizures in KA-induced chronic seizure rats. (**a**) Heatmap of daily recorded total spontaneous seizures. V1–6 = Vehicle group, G1–6 = Ganaxolone 7.6 mg/kg treatment group, and L1–6 = LPM682000012 3.6 mg/kg treatment group. (**b**) The daily recorded average number of seizures. (**c**) Average seizure score detected each day. (**d**) Average total durations detected each day. Results are indicated as mean ± SEM, n = 6. ### *p* < 0.001, # *p* < 0.05 vs. Ganaxolone group, and *** *p* < 0.001, * *p* < 0.05 vs. Vehicle group. ● means Vehicle group, ■ means Ganaxolone treatment group, ▲ means LPM682000012 treatment group.

This present study investigated LPM682000012’s anti-epileptic potential using the KA-induced chronic spontaneous seizure model, which has a high similarity to clinical chronic epilepsy syndrome [19]. The data indicated that LPM682000012 markedly alleviated the number of seizures per day (*p* < 0.001), seizure score detected each day (*p* < 0.001), and the total seizure duration per day (*p* < 0.001) compared to the Ganaxolone treatment (Figure 4a–d). Overall, it was observed that LPM682000012 attenuated seizure severity in the KA-induced chronic epilepsy model.

### 2.4. LPM682000012 Ameliorated Neuronal Loss in KA-Induced Chronic Epileptic Rats

**Figure 5:**
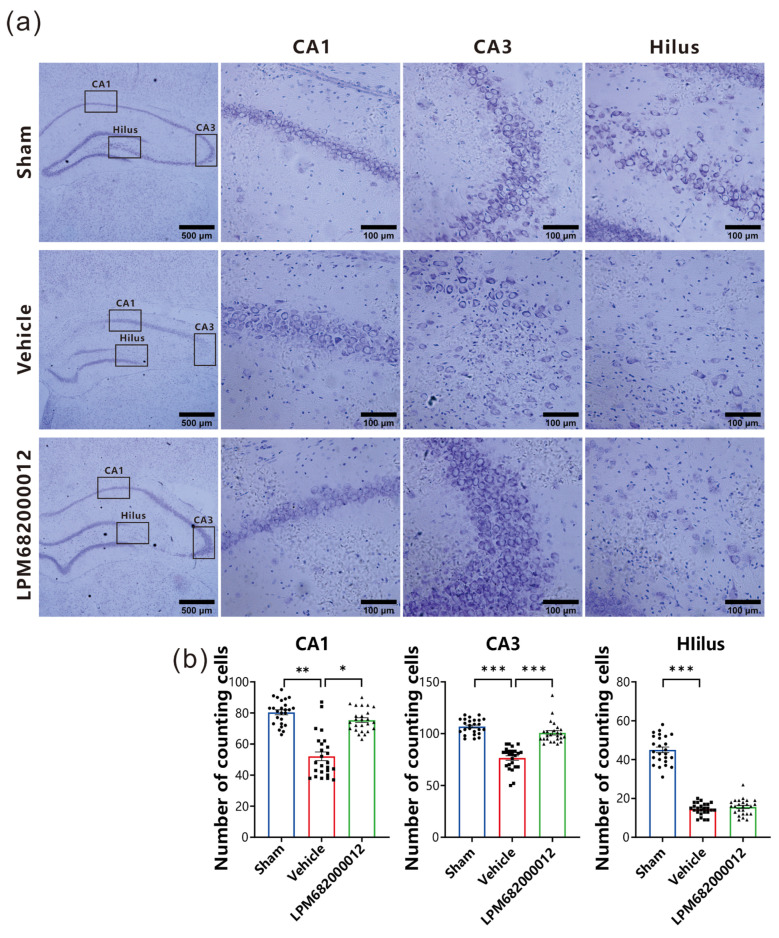
Nissl staining revealed neuroprotection of LPM682000012 treatment. (**a**) Representative micrographs of Nissl staining. (**b**) Pyramidal cell counts in the hippocampal CA1, Hilus, and CA3 regions. Results are indicated as mean ± SEM, n = 25. *** *p* < 0.001, ** *p* < 0.01, * *p* < 0.05 vs. Vehicle group. ● means Sham group, ■ means Vehicle group, ▲ means LPM682000012 treatment group.

To elucidate LPM682000012’s potential neuroprotective effect against KA-induced neurotoxicity, intact neurons in the hippocampal CA1, hilus, and CA3 regions were quantified and analyzed by Nissl staining. The Vehicle-treated rats indicated necrosis and neuronal cell death in the CA1, hilus, and CA3 regions (Figure 5a). Furthermore, there is a significant amount of neuronal loss that has been found in CA1 (*p* < 0.01), CA3 (*p* < 0.001), and hilus (*p* < 0.001) of the Vehicle-treated group compared with the Sham group (Figure 5b). However, repeated LPM682000012 (3.6 mg/kg) or Ganaxolone (7.6 mg/kg) treatment notably attenuated KA-induced loss of neurons in both CA3 (*p* < 0.001) and CA1 (*p* < 0.05) (Figure 5b) regions but not the hilus region. Altogether, these data revealed that LPM682000012 has a neuroprotective effect on hippocampal neurons of the KA-induced epileptic rats.

### 2.5. LPM682000012 Increased the Density of Hippocampal CA1 Dendritic Spines 

**Figure 6:**
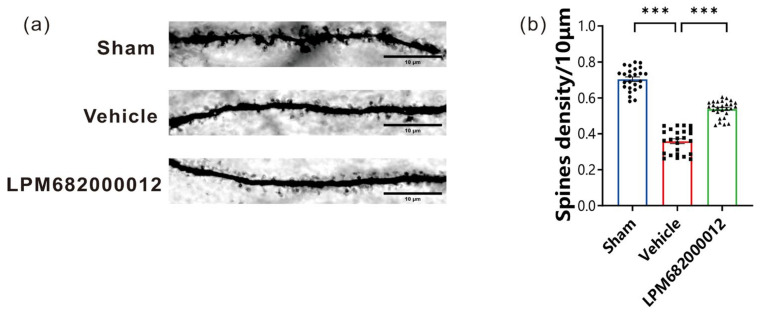
LPM682000012 decreased the density of hippocampal CA1 dendritic spines. (**a**) Golgi staining representative images indicated the differences in the hippocampus dendritic spine morphology between each group. Scale bar = 10 μm. (**b**) Total spine density in the hippocampus. Results are indicated as mean ± SEM, n = 25, *** *p* < 0.001 vs. Vehicle group. ● means Sham group, ■ means Vehicle group, ▲ means LPM682000012 treatment group.

In this research study, Golgi staining was carried out to elucidate the morphological changes in the rat’s hippocampal CA1 region dendritic spines after 8 weeks of KA injection. It was revealed that compared to the Sham group, the total density of dendritic spines was notably decreased in rats after KA injection (*p* < 0.001); however, this effect was prevented by repeated LPM682000012 (3.6 mg/kg) (*p* < 0.001) treatment (Figure 6a,b), suggesting that LPM682000012 can inhibit synaptic remodeling.

### 2.6. Transcriptomics Analysis of Differential mRNA Expression in the Hippocampus of KA-Induced Chronic Epileptic Rats 

**Figure 7:**
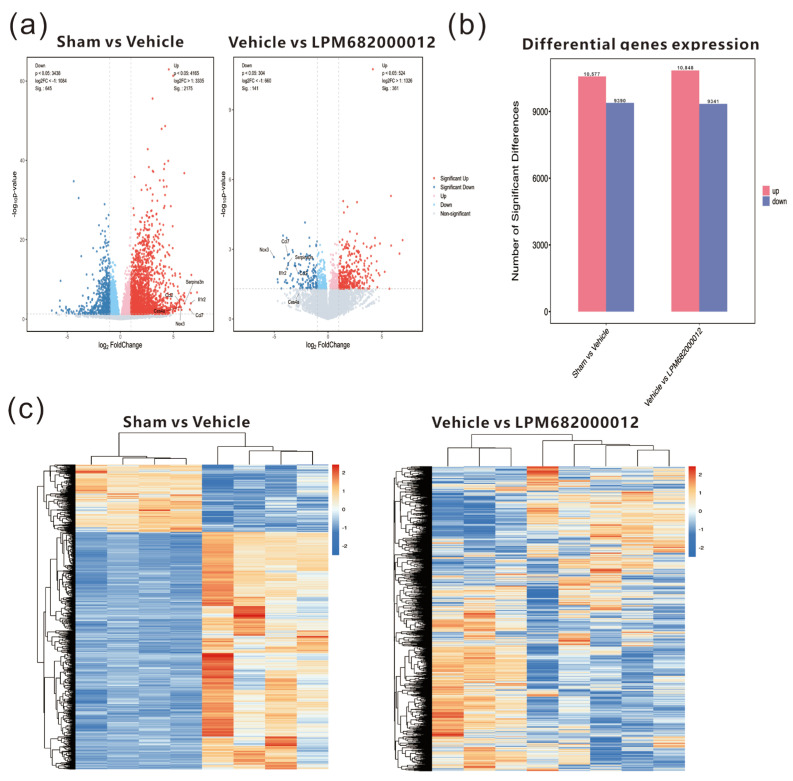
Transcriptomics analysis of hippocampal tissues from Sham-, Vehicle-, or LPM682000012-treated rats. (**a**) RNA sequencing volcano plot. (**b**) Bar graphs of differential expression of genes in the hippocampus of Vehicle-treated vs. Sham-treated and LPM682000012-treated vs. Vehicle-treated rats. (**c**) Heatmap of the differential mRNA expression clustering (top 1000) in the rat’s hippocampus. Orange = up-regulated genes (*p* < 0.05 and fold change > 2), and Blue = down-regulated genes (*p* < 0.05 and fold change < 0.5).

To further examine the potential antiepileptic mechanism of LPM682000012, transcriptome analysis of KA-induced chronic epileptic rats’ hippocampal tissues was performed. The results indicated 10,577 up-regulated and 9390 down-regulated genes in the Sham-treated vs. Vehicle-treated rats, whereas 10,848 up-regulated and 9341 down-regulated genes were identified in the Vehicle-treated vs. LPM682000012 (3.6 mg/kg)-treated rats (Figure 7a,b). The heatmap indicates the clustering of transcript sequences (top 1000) in the hippocampus; there were marked differences between the groups (Figure 7c). Moreover, the two sets of differential genes were further assessed to select the up-regulated genes in the Sham-treated vs. Vehicle-treated rats and the down-regulated genes in the Vehicle-treated vs. LPM682000012-treated rats. Moreover, the six most differentially expressed genes (DEGs) were also identified (Table 2).

### 2.7. qRT-PCR Analysis of Hippocampal Tissue’s DEG mRNA Levels

**Figure 8:**
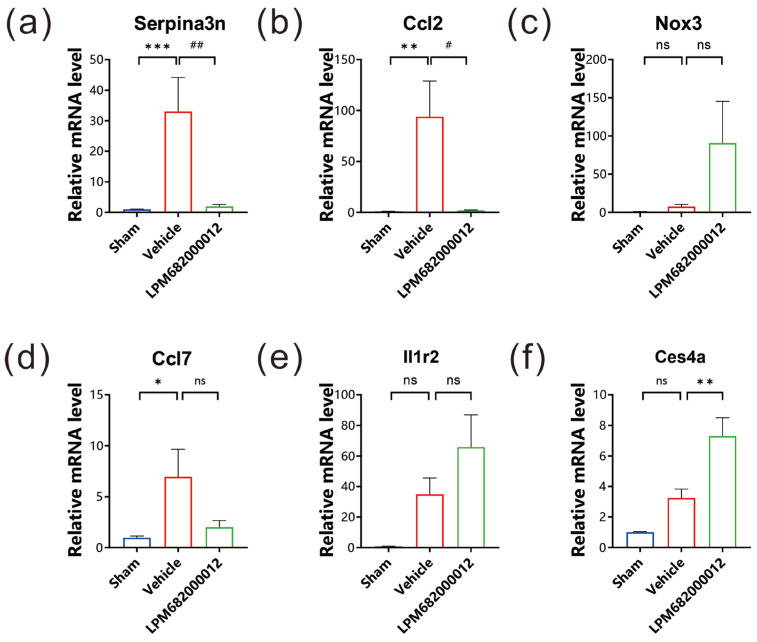
mRNA levels were evaluated via qRT-PCR. The expression levels of (**a**) *Serpina3n*, (**b**) *Ccl2*, (**c**) *Nox3*, (**d**) *Ccl7*, (**e**) *Il1r2*, and (**f**) *Ces4a* in each group. Results are indicated as mean ± SEM, n = 6. ns means not significant, *** *p* < 0.001, ** *p* < 0.01, * *p* < 0.05, ## *p* < 0.01, # *p* < 0.05 vs. Vehicle group.

To evaluate the mRNA levels of the six identified DEGs, qRT-PCR analysis was carried out using the hippocampal tissues of KA-induced chronic epileptic rats. It was observed that three genes, *Serpina3n*, *Ccl2* and *Ccl7*, had substantially higher expression in Vehicle-treated rats (*Serpina3n*: *p* < 0.001, *Ccl2*: *p* < 0.01, *Ccl7*: *p* < 0.05), but only *Serpina3n* and *Ccl2* notably reduced expression in LPM682000012 (3.6 mg/kg)-treated rats relative to the Vehicle-treated rats (*Serpina3n*: *p* < 0.01, *Ccl2*: *p* < 0.05) (Figure 8a,b,d). The *Nox3*, *Il1r2*, and *Ces4a* had no significant difference between Sham and Vehicle-treated rats (Figure 8c,e,f).

### 2.8. Proteomics Analysis of Differential Protein Expression in the Hippocampus of KA-Induced Chronic Epileptic Rats

**Figure 9:**
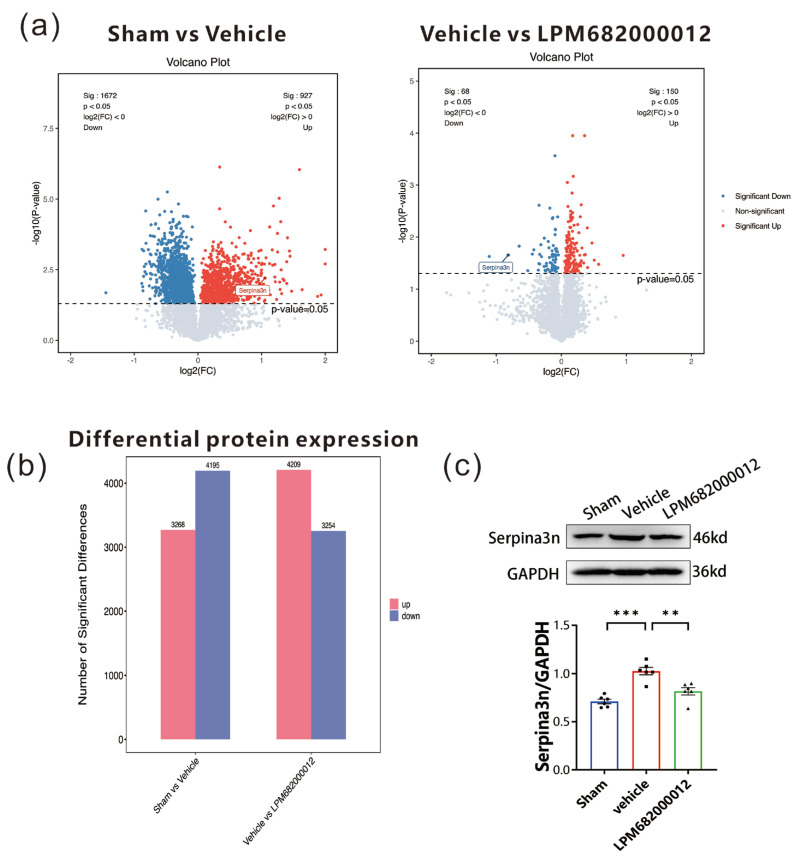
Proteomics analysis of hippocampal tissues from Sham-, Vehicle-, or LPM682000012-treated rats. (**a**) proteomics volcano plot. (**b**) Bar graphs represent differential protein expression levels in the hippocampus of Vehicle-treated vs. Sham-treated rats and LPM682000012-treated vs. Vehicle-treated rats. (**c**) Changes in protein levels of *Serpina3n* detected by Western blot. Results are represented as mean ± SEM, n = 6, *** *p* < 0.001 and ** *p* < 0.01 vs. Vehicle group. ● means Sham group, ■ means Vehicle group, ▲ means LPM682000012 treatment group.

To elucidate the association between identified DEGs and epilepsy-related proteins, proteomic analysis was carried out using the hippocampal tissues from KA-induced chronic epileptic rats. The data revealed 3268 up-regulated and 4195 down-regulated proteins in the Sham-treated vs. Vehicle-treated rats. Furthermore, 4209 up-regulated and 3254 down-regulated proteins in the Vehicle-treated vs. LPM682000012 (3.6 mg/kg)-treated rats (Figure 9a,b). Based on the identified DEGs, the two sets of differential proteins were screened, which revealed that only *Serpina3n* was up-regulated in the Sham-treated vs. Vehicle-treated rats and down-regulated in the Vehicle-treated vs. LPM682000012 (3.6 mg/kg)-treated rats (Figure 9a). The hippocampal *Serpina3n* protein expression levels were evaluated by Western blot analysis, which revealed markedly enhanced expression in the Vehicle-treated group compared to the Sham group (*p* < 0.001), whereas LPM682000012 treatment notably reduced *Serpina3n* expression level (*p* < 0.001) (Figure 9c).

### 2.9. Serpina3n Stimulates the NF-κB Signaling Pathway in Epileptic Rats

**Figure 10:**
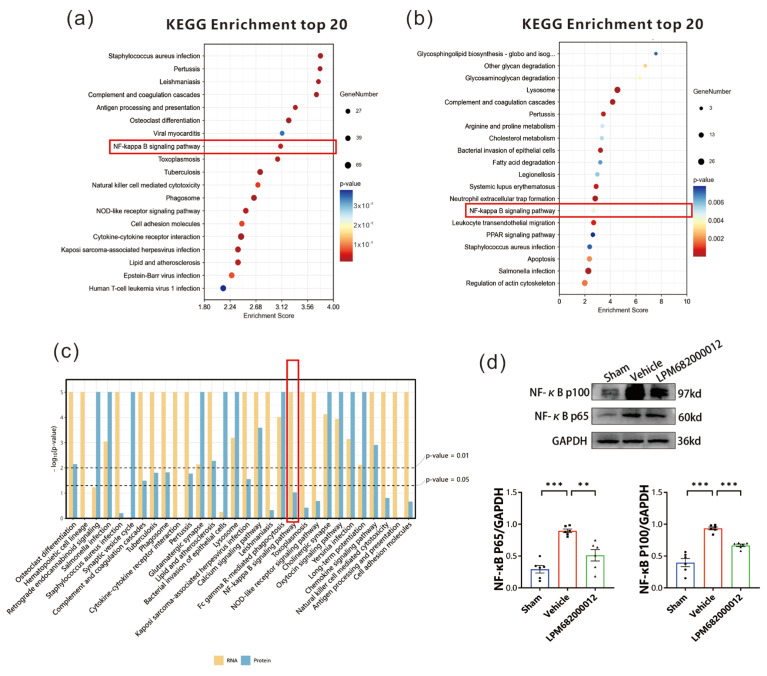
NF-κB signaling pathway in epilepsy rats. (**a**) Transcriptomic KEGG enrichment analysis. Dot size = total genes in each KEGG pathway. X-axis = significant gene’s enrichment score over the total genes in a given pathway. (**b**) Proteomics KEGG enrichment analysis. Dot size = total genes in each KEGG pathway. X-axis = significant protein’s enrichment score over the total proteins in a given pathway. (**c**) KEGG enrichment analysis for joint analysis. (**d**) Changes in NF-κB (p65) and NF-κB (p100) protein levels were detected by Western blot. Results are given as mean ± SEM, n = 6, *** *p* < 0.001 and ** *p* < 0.01 vs. Vehicle group. ● means Sham group, ■ means Vehicle group, ▲ means LPM682000012 treatment group.

To elucidate how *Serpina3n* is involved in epilepsy in rats, the transcriptomics and proteomics KEGG enrichment pathways were screened. Transcriptomic KEGG pathway analysis indicated multiple notably enriched inflammatory pathways, including the NF-κB and NOD-like receptor signaling pathways, where the NF-κB pathway had a higher enrichment score (Figure 10a). Furthermore, the proteomics KEGG enrichment analysis validated increased enrichment of the NF-κB signaling pathway (Figure 10b). Thus, the NF-κB signaling pathway was observed as the common inflammatory pathway in joint analysis (Figure 10c). Western blotting revealed that in Vehicle-treated KA-induced chronic epilepsy rats, the NF-κB (p65) and NF-κB (p100) protein levels were increased in the hippocampus (*p* < 0.001), whereas these effects were reversed by the LPM682000012 treatment [NF-κB (p65): *p* < 0.01, NF-κB (p100): *p* < 0.001] (Figure 10d).

## 3. Discussion

The currently available pharmacotherapies cannot effectively control epilepsy [1]. NASs are a promising resource for novel ASDs. This study revealed that LPM682000012, a novel synthetic neuroactive steroid PAM of extra-synaptic and synaptic GABA_A_ receptors, could alleviate PTZ-induced acute epileptic seizure with a higher safety margin than Ganaxolone. Furthermore, LPM682000012 also inhibited KA-induced chronic spontaneous seizures in rats and reversed the loss of neuronal bodies and hippocampus synaptic remodeling in these rats. Mechanistically, LPM682000012 activated the synaptic and extra-synaptic GABA_A_ receptors and improved KA-induced alterations in the neuronal bodies and synaptic structure, potentially by downregulating the *Serpina3n*/NF-κB signaling pathway. This suggests that LPM682000012 is a promising anti-seizure agent with a lower sedative effect than Ganaxolone that can be employed for the pharmacotherapy of epilepsy.

GABA_A_ receptors are the most critical component in the GABA system, modulating neuronal and seizure activity. When GABA stimulates synaptic GABA_A_ receptors, they predominantly regulate rapid phasic suppression, while extra-synaptic GABA_A_ receptors primarily modulate tonic inhibition [20]. NASs or other synthetic derivatives are increasingly being considered for epilepsy treatment [21,22]. There are several differences between NASs and benzodiazepines [23]. NASs preferential binds extra-synaptic GABA_A_ receptors as well as can also bind and regulate synaptic GABA_A_ receptors. Therefore, NAS has an advantage as GABA_A_ receptor-positive allosteric modulators in epilepsy over benzodiazepines, which only regulate synaptic GABA_A_ receptors [24,25,26].

Ganaxolone, a methyl analog of allopregnanolone, has been approved in the USA for treating CDD-associated seizures in patients with age ≥ 2 years [15]. It is currently under phase III clinical trial for refractory status epilepticus (RSE) by Marinus Pharmaceuticals [27]. However, Ganaxolone has certain clinical limitations and adverse reactions, such as somnolence and sedation, with incidence rates of 38% and 6%, respectively. Sedation and somnolence are primarily observed in the early stages of the treatment and are generally dose-related [15]. Here, a sedation assay was carried out in rats, including the evaluation of LORR and the loss of the toe-pinch reflex. It was observed that both LPM682000012 and Ganaxolone can produce profound behavioral sedation at relatively large doses.

This present study also evaluated the anti-seizure activities of sub-sedative doses of LPM682000012 and Ganaxolone in the PTZ-induced GSs rat model. Both LPM682000012 and Ganaxolone demonstrated dose-dependent and effective anti-seizure activities, with LPM682000012 indicating a higher safety margin. KA is a glutamate receptor agonist that can induce status epilepticus, which promotes chronic, spontaneous, and recurrent seizures [28,29]. Intrahippocampal KA injection has been used to establish an epilepsy model, which is a commonly employed difficult-to-treat seizure model for investigating the neuronal mechanisms of epilepsy and anti-seizure drug development [19]. Here, it was found that LPM682000012 was more efficacious than Ganaxolone in protecting against the development of spontaneous seizures in the KA-induced epilepsy model. Altogether, these in vivo results indicated that LPM682000012 has broad-spectrum anti-epileptic activity, has a better safety margin, is more potent, and is sometimes more effective than Ganaxolone.

To investigate the potential pathways by which LPM682000012 could produce antiepileptic effects in addition to modulating extra-synaptic and synaptic GABA_A_ receptors, the researchers evaluated the effects of LPM682000012 on KA-induced synaptic plasticity and neuronal deficits. A decrease in the number of neurons is crucially related to epilepsy recurrence. Hippocampal neuronal cell death is considered a frequent pathological hallmark of epilepsy. The literature indicates that in chronic epilepsy, GABAergic inhibitory interneurons in the hippocampus and CA3 region are reduced, which causes epilepsy recurrence [30]. Furthermore, KA induces significant neuronal loss and impairs neuronal structures in the hippocampal CA1, CA3, and hilus [31,32,33]. In our study, the Vehicle-treated rats indicated necrosis and neuronal cell death in the CA1, hilus, and CA3 regions and were reversed by the LPM682000012 treatment. Moreover, prolonged or recurrent epileptic seizures promote neuronal damage [34]. Synaptic plasticity, the activity-dependent alteration in neuronal connectivity strength, is essential for neuronal inhibition and excitation [35]. 

In addition, the pathogenesis of epilepsy includes synaptic remodeling [36]. Epileptogenesis is closely related to synaptic remodeling. It has been observed that alterations in the number and distribution of dendritic spines are directly associated with epileptogenesis and seizures in chronic epilepsy rats and drug-resistant epilepsy patients [37,38]. Dendritic spines are thin, small, specialized neuronal dendrite protrusions that are mainly located in excitatory synapses [39]. Dendritic spines mostly receive and integrate excitatory synaptic inputs from the hippocampus and mammalian cortex, directly affecting seizures and neuronal excitability under pathological conditions [40]. Since dendritic spines are a crucial component of excitatory synapses, their density and morphology are critically linked with synaptic plasticity [39]. In many studies of Golgi staining experiments associated with KA-induced chronic epilepsy, it was shown that spine changes were mainly associated with CA1 [41,42]. The studies of dendritic spines in CA3 and hilus are controversial [41,43,44,45]. Here, dendritic spine density was observed in the hippocampal CA1 neurons of KA-induced chronic epileptic rats; however, LPM682000012 alleviated these changes. This suggests that preventing deleterious neuronal loss and synaptic plasticity maladaptation may be the morphological substrate of LPM682000012 underlying its anti-epilepsy activity. 

Transcriptomics and proteomics analyses of the KA-induced chronic epileptic rat’s hippocampal tissues were conducted to assess how LPM682000012-induced anti-epileptic effects. An aberrant transcript profile was observed, and six most DEGs were identified, which were up-regulated in the Sham vs. Vehicle treatment groups and down-regulated in the Vehicle vs. LPM682000012 treatment groups. qRT-PCR assessed the mRNA levels of these DEGs and indicated that the LPM682000012 markedly decreased *Serpina3n* and *Ccl2* mRNA levels. Moreover, the transcriptomics and proteomics analysis showed that the NF-κB pathway was activated in the hippocampus of KA-induced chronic epileptic rats based on. The studies of NF-κB showed that P65 and P100 are the more detected indicators of this pathway and are sufficient to indicate changes in the NF-κB pathway [46,47], especially P65 is associated with both KA-induced chronic epilepsy and *Serpina3n* [33]. Western blot was performed to examine the *Serpina3n* and NF-κB protein expression levels and revealed that their levels were enhanced in the hippocampus of epileptic rats, which was reversed by LPM682000012 treatment. Overall, these data revealed that the *Serpina3n*/NF-κB pathway might be crucially associated with the anti-seizure effect of LPM682000012.

The intrinsic brain neuroinflammation response comprises innate immune mechanisms that stimulate neuronal, glial, and microvascular systems [48]. Brain inflammation has been found to initiate and perpetuate seizures in various epilepsies [49]. After activation, microglia can stimulate different proinflammatory factors to promote neuroinflammation and even neuronal loss [50]. Peripheral immune cell infiltration into the nigrostriatal system can result in neuronal loss and damage [51]. The literature has indicated that enhanced expression of inflammatory molecules can reduce various neural plasticity indices, such as membrane excitability, neurogenesis, synaptic transmission, dendritic spine density, and pyramidal neuron plasticity [52,53]. Thus, neuroinflammation is actively involved in neuronal loss and synaptic remodeling.

*Serpina3n* encodes anti-chymotrypsin, a widely expressed serpin superfamily member. It has been observed to be related to the positive feedback with proinflammatory cytokines [54,55,56]. In addition, studies have indicated that *Serpina3n* induces proinflammatory cytokine secretion via the NF-κB signaling pathway, which can be occluded by the NF-κB inhibitor BAY11 [54]. During inflammation, NF-κB has been observed to release and modulate different inflammatory cytokines [57,58,59], which are linked with the pathogenesis of various central nervous system disorders, such as epilepsy [60].

The studies of NF-κB showed that P65 and P100 are the more detected indicators of this pathway and are sufficient to indicate changes in the NF-κB pathway [46,47], especially P65 is associated with both KA-induced chronic epilepsy and *Serpina3n* [33].

**Figure 11:**
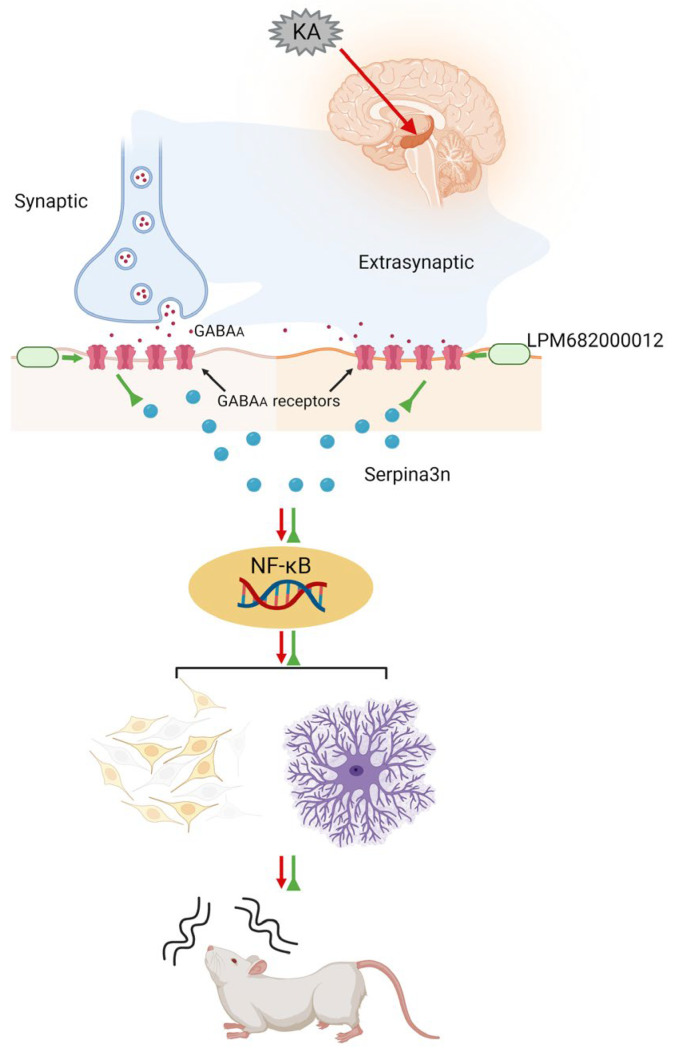
Schematic model showing that LPM682000012 activated the synaptic and extra-synaptic GABA_A_ receptors and ameliorated KA-induced neuronal loss and synaptic remodeling, presumably via downregulating the *Serpina3n*/NF-κB signaling pathway.

Much literature has indicated that activated GABA_A_ receptors can inhibit the progression of various inflammation-related diseases. In addition to their direct activation by GABA, their activation is modulated by steroid-PAMs in response to neuroinflammation [61], such as Ganaxolone, which enhances GABA_A_ receptor activation [62]. Moreover, Ganaxolone reduces neonatal seizures partially by reducing neuroinflammation within the thalamus [63]. GABA_A_ receptor activation has been observed to inhibit the NF-κB signaling [64]. Based on this information, it was postulated that LPM682000012 promotes an anti-epileptic effect by directly activating the synaptic and extra-synaptic GABA_A_ receptors, which alleviates KA-induced neuronal loss and synaptic remodeling, a process potentially mediated by downregulating the *Serpina3n*/NF-κB signaling pathway (Figure 11).

## 4. Materials and Methods

### 4.1. Animals

Sprague–Dawley (SD) (adult, weight = 180–200 g, male) rats were procured from the Pengyue Laboratory Animal Supplies Inc. (Jinan, China) and kept in a 12 h dark and 12 h light cycle at 21–23 °C, 40–60% humidity in a certified animal facility with libitum water and chow access. Before the behavioral analyses, all the rats were acclimatized for at least 2 days to the test environment. Furthermore, Animal studies are reported in compliance with the ARRIVE guidelines [65]. All in vivo analyses were authorized by the Yantai University Laboratory Animal Care and Use Committee (protocol #: YTU20221123). The researchers performing the behavioral tests were blinded to the treatment conditions.

### 4.2. Drugs

LPM682000012 (>95% pure, white powder) was acquired from the Key Laboratory of Molecular Pharmacology and Drug Evaluation (Yantai University). BioChemPartner (Shanghai, China) provided Ganaxolone (>99.8% pure, white powder). Ganaxolone and LPM682000012 suspensions were prepared by dissolving them in 10% DMSO and 90% pre-prepared sulfobutylether-beta-cyclodextrin (30%). Vehicle-treated rats were administered by the Vehicle (same solvent as the drug). The freshly prepared drug solutions were administered daily.

### 4.3. Sedative Effect 

For sedative effects, prior to the formal experiment we conducted a preliminary test. To assess the sedative effects, rats (3/group) were intragastric gavaged (i.g.) with LPM682000012 (40, 35, 30, and 28 mg/kg) or Ganaxolone (80, 64, 50, and 40 mg/kg) and observed for the sedative effect for 3 h and scored as follows: 0 = no sedation, awake, and no alteration in observed behavior or locomotion; 1 = light sedation, intact righting reflex, and slowed movement; 2 = medium sedation and loss of righting reflex (LORR), but recoverable in 1 min; 3 = full sedation and LORR unrecoverable in 1 min, but responsive to toe-pinch reflex; 4 = anesthesia and loss of toe-pinch reflex. LPM682000012 at the dose higher than 30 mg/kg induced death of rats; Ganaxolone at the dose higher than 40 mg/kg also induced death of rats (Supplementary Materials).

To assess the sedative effects, rats (10/group) were intragastric gavaged (i.g.) with LPM682000012 (19.7, 21.9, 24.3, 27, and 30 mg/kg) or Ganaxolone (26.2, 29.2, 32, 36, and 40 mg/kg) and observed for the sedative effect for 3 h. Experienced investigator blinded to protocol performed scoring.

### 4.4. Pentylenetetrazol (PTZ) Seizure Model and Behavioral Assessment of Seizure Severity

**Figure 12:**
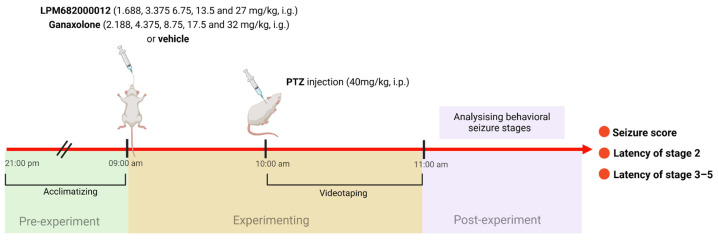
A scheme of PTZ-induced seizure model.

The PTZ model was performed in the previous study [66]. The maximum doses of LPM682000012 and Ganaxolone in the PTZ model were the doses at which rats do not experience full sedation. Rats were randomly assigned into 11 groups: Vehicle (10% DMSO and 90% pre-prepared 30% sulfobutylether-beta-cyclodextrin), Ganaxolone (2.188, 4.375, 8.75, 17.5, and 32 mg/kg; i.g.), and LPM682000012 (1.688, 3.375, 6.75, 13.5, and 27 mg/kg; i.g.) treatment groups. After 1 h of Ganaxolone [67], 2 h of LPM682000012 [16], or Vehicle administration, rats were administered with PTZ (40 mg/kg; i.p.) and observed for 1 h for seizure activity. Based on the Racine (1972) protocol [68], the behavioral seizure’s severity was scored: stage 5 (falling and jumping with bilateral forelimb clonus), stage 4 (bilateral forelimb clonus and rearing), stage 3 (unilateral forelimb clonus), stage 2 (nodding), and stage 1 (ear and facial movement). An investigator blinded to the rat’s categorization graded the seizure severities (Figure 12).

### 4.5. Kainic Acid (KA)-Induced Chronic Epilepsy Model

**Figure 13:**
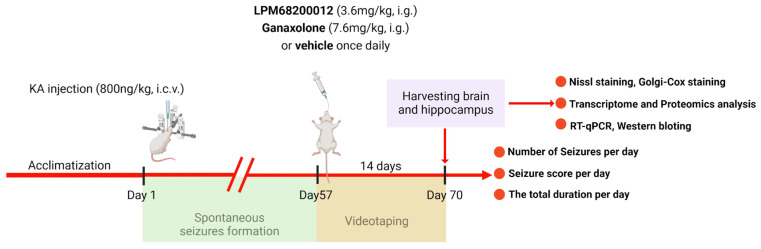
A scheme of KA-induced chronic epilepsy model.

Isoflurane (2% maintenance and 4% induction) was employed for anesthetizing rats to fix their heads on a stereotactic frame. The chronic epilepsy model was established using KA based on the previous reports [69,70]. Briefly, the right of the rat’s hippocampus was slowly and unilaterally injected with 800 nL KA (1.5 ng/nL) at the speed of 200 nL min^−1^ as follows: ML—2.5 mm, AP—3.5 mm, and DV—3.5 mm. In the acute PTZ model, we have calculated the ED_50_ for LPM and Ganaxolone, then we chose the ED_50_ of the latency of stages 3–5 to determine whether LPM and Ganaxolone are effective in the model with KA. After 2 months of KA injection (spontaneous seizure period), rats were administered 7.6 mg/kg Ganaxolone (i.g.) or 3.6 mg/kg LPM682000012 (i.g.) for 14 days once daily [71] (Figure 13). Used the videotaping system to test rats 24 h per day for 14 days without interruption. The number of seizures, seizure score, and total durations were recorded by investigators blinded to the rat’s categorization.

### 4.6. Nissl Staining

The rat’s dorsal hippocampus (1.80–3.0 mm posterior to bregma) coronal sections (25 μm) were collected, dyed with crystal violet (Cat#C0117, Beyotime Institute of Biotechnology, Shanghai, China) per the kit’s guide, stained for 10 min with 1% toluidine blue at 37 °C, washed twice with distilled water (each time for 2 min), dehydrated twice using 95% ethanol (each time for 2 min), clarified for 5 min in xylene, and covers-lipped with neutral balsam. The total number of cells in the hippocampal CA1, hilus, and CA3 regions were counted from 3 non-overlapping fields of each section (400×; Carl Zeiss Axio Scope A1 microscopy, Zeiss, Oberschon, Germany) and analyzed via a computer-aided image analysis system (ZEN 2.3 Lite, Zeiss, Oberschon, Germany). Pathologists blinded to the treatments performed these procedures to avoid any bias in cell quantification. The same-sized positive cells in fields of view were manually counted via the Image J2 (National Institutes of Health, Bethesda, MD, USA). For this analysis, brain tissues were acquired from 5 rats/groups; from each rate, 5 histological sections were prepared, and from each section, 5 different fields were analyzed. 

### 4.7. Golgi–Cox Staining

The protocol of the FD rapid Golgi Stain kit (FD Neurotechnologies, Columbia, SC, USA) and previously described methods were employed for Golgi–Cox staining [72,73]. Briefly, rats were injected with pentobarbital sodium (35 mg/kg, i.p.) for anesthetization, intracardially perfused with physiological saline, and sacrificed by decapitation. The rat’s hippocampal tissues were immediately sampled and treated with the impregnation solution (solutions A and B dissolved in equal volume) at ambient temperature in the dark for 24 h. The AB mixture was then refreshed, tissues were incubated for 2 to 3 weeks, and treated with C solution, which was refreshed after 24 h and then soaked for 7 more days. The dry ice–isopentane method was employed for quick freezing of the tissues, which were then immersed in wax, sliced (140 μm thick) with a cryostat, fixed on gelatin-laminated glass slides, dyed with D and E solutions, dehydrated in gradient ethanol, cleared using xylene, and mounted with Eukitt (O. Kinde) mounting medium. Carl Zeiss Axio Scope A1 microscope (Zeiss, Oberschon, Germany) with ZEN 2.3 Lite (Zeiss, Oberschon, Germany) was employed for imaging. Experienced investigators blinded to the treatments and analyzed the images via the Image J software (National Institutes of Health, Bethesda, MD, USA). In hippocampal neurons, the number of spines/40 μm dendrite segments was counted. For this analysis, brain tissues were acquired from 5 rats/groups; from each rate, 5 histological sections were prepared, and from each section, 5 different fields were analyzed.

### 4.8. Transcriptome and Proteomics Analyses

These analyses were carried out using OE Biotech Co., Ltd. (Shanghai, China). Briefly, the whole RNA was isolated with the help of TRIzol, per the kit’s guide. Then, a NanoDrop 2000 spectrophotometer (Applied Biosystems, Carlsbad, CA, USA) was utilized for RNA purity and quantification analyses. Furthermore, the Agilent 2100 Bioanalyzer (Agilent Technologies, Santa Clara, CA, USA) was utilized to evaluate RNA integrity before the construction of libraries by following the methods provided in the VAHTS Universal V6 RNA-seq Library Prep Kit (Vazyme Biotech, Nanjing China). 

Total tissue protein was isolated via the standard phenol method, quantified by bicinchoninic acid assay, digested using DTT, labeled with TMT, and subjected to RP separation by 1100 HPLC (Agilent) System with the help of an Agilent Zorbax Extend RP column (5 μm, 150 mm × 2.1 mm). A Nanospray Flex source (Thermo, Waltham, MA, USA) equipped Q Exactive HF mass spectrometer (Thermo, USA) was utilized for all the analyses, and the libraries were established via ProteomeDiscoverer 2.4.1.15 (ThermoFisher Scientific) per the kit’s protocol.

### 4.9. Quantitative Real-Time Polymerase Chain Reaction (qRT-PCR)

qRT-PCR was performed as in the previous study [74]. Pentobarbital sodium (35 mg/kg, i.p.) was employed as an anesthetic drug; rats were intracardially perfused with physiological saline; their whole brains were dissected; and the hippocampus was sampled for an mRNA test. The whole RNA from the samples was harvested via an RNA extraction kit and used for cDNA synthesis using a reverse transcription kit, which was then quantified via qRT-PCR. The SYBR Green method with primer OligodT was employed for qRT-PCR per the kit’s guide (Sangong, Shanghai, China). Table 3 lists the primers used in this analysis.

The qRT-PCR protocol included 10 μL reaction mix [0.4 μL reverse and forward primer (10 nM), 0.2 μL ROX Reference Dye II, 5 μL SYBR Green qRT-PCR Mix, 0.5 μL cDNA, and 3.9 μL RNase-free H_2_O]. For amplification, Power SYBR Green PCR Master Mix was tested in the Fast 7500 Real-Time PCR System (Applied Biosystems, Carlsbad, CA, USA). The experiment was carried out in triplicate with the following parameters: 1 cycle; 10 min at 95 °C, 40 cycles; 15 s at 95 °C, and 60 s at 60 °C, melting cycle; 15 s at 95 °C, 60 s at 60 °C, and 30 s at 95 °C, followed by cooling for 30 s at 37 °C. The gene expressions were normalized to those of the *GAPDH* gene. The difference in gene expressions between groups was determined by the ΔΔCt, while relative fold changes were assessed by the 2^ΔΔCt^ method.

### 4.10. Western Blotting

Western blotting was performed as in the previous study [74]. The dissected hippocampal tissues were homogenized in RIPA buffer on ice for 30 min, and amygdala samples were centrifuged for 20 min at 13,400× *g* to collect supernatants. The proteins in the samples were quantified via a bicinchoninic acid (BCA) Protein Assay kit (Beyotime, Shanghai, China). Then, 50 μg of the quantified protein was resolved on a 4–20% SDS-PAGE gel, transferred to polyvinylidene difluoride membranes, occluded for 2 h in 5% non-fat milk (*m*/*v*), co-treated overnight with primary antibodies: rabbit anti-*Serpina3n* (Cat# YT5391), rabbit anti-NFκB-P65 (Cat# YT3108), rabbit anti-NFκB-P100 (Cat# YT3093) (all 1:1000 diluted, acquired from ImmunoWay Biotechnology Company, Barksdale Professional Center, Newark, NJ, USA) and mouse anti-*GAPDH* (1:1000, Beyotime Cat# AF0006) at 4 °C, washed thrice with TBST-Tween 20, and probed with the secondary goat anti-rabbit HRP (Cat# A020) and goat anti-rat HRP (Cat# A0216) (both 1:2000 diluted, acquired from Beyotime) antibodies for 1 h. The membranes were then reacted with ChampChemi 610 (Sagecreation, Beijing, China) with Sage Capture Pro 5.0 software (Sagecreation, Beijing, China). The Image J2 software was employed to quantify signal intensities, which were normalized to the corresponding *GAPDH* band.

### 4.11. Statistical Analysis

All the statistical assessments were carried out via GraphPad Prism v 9.0 (La Jolla, CA, USA) and SPSS for Windows^®^ version 21.0 (Chicago, IL, USA), and the data were indicated as means ± SEM. The data were assessed via one-way ANOVA with Bonferroni multiple comparison correction. Statistically, the *p*-value of <0.05 was deemed as the significance threshold. 

## 5. Conclusions

In summary, this research study investigated the potential anti-epileptic effects of LPM682000012 in different epilepsy models and found that LPM682000012 has a broad-spectrum antiepileptic efficacy and better safety profile than Ganaxolone. Mechanistically, LPM682000012 was found to activate the synaptic and extra-synaptic GABA_A_ receptors and ameliorate KA-induced neuronal loss and synaptic remodeling by downregulating the *Serpina3n*/NF-κB signaling pathway. This study validated that LPM682000012 is a novel and promising neurosteroid PAM with therapeutic potential for epilepsy and warrants further investigation.

## Figures and Tables

**Table 1:** Safety margins of LPM682000012 and Ganaxolone.

	Seizure Score	The Latency of Stage 2	The Latency of Stage 3–5
LPM682000012	5.7	27.0	7.5
Ganaxolone	3.3	2.7	4.2

Safety margin = dose for inducing loss of consciousness/ED_50_.

**Table 2:** Log2FoldChange of the six DEGs identified through transcriptomics analysis.

Gene Name	Log2FordChange (Sham vs. Vehicle)	Log2FordChange (Vehicle vs. LPM682000012)
*Il1r2*	6.64	−3.71
*Ccl7*	6.54	−3.73
*Serpina3n*	6.04	−3.70
*Nox3*	5.71	−5.03
*Ccl2*	5.56	−3.07
*Ces4a*	5.51	−2.69

**Table 3:** Set of primers employed for qRT-PCR.

Gene	Forward	Reverse
*GAPDH*	GGTCGGAGTCAACGGATTTG	ATGAGCCCCAGCCTTCTCCAT
*Il1r2*	ACTACGTGGAAGTGTCGCTG	ATATCGCCCCCACAACCAAG
*Ccl7*	GGGACCAATTCATCCACTTGC	TCAACCCACTTCTGATGGGC
*Serpina3n*	ATGACCCGCCTTGTGACTCTG	CCCCTTGTCTTGGTCTTCATGG
*Nox3*	CCCTGTGGTCTTGTATGCGT	AAAGATGTACTGTCCGGGCG
*Ccl2*	TGTTCACAGTTGCTGCCTGTA	TCTTGTAGTTCTCCAGCCGAC
*Ces4a*	AAGTGATGGAGCAGACCACG	TGTCAAAGCGAGGCCAGTAG

## Data Availability

Some or all data generated or analyzed during this study are included in this published article.

## Supplementary Materials

The following supporting information can be downloaded at: https://www.mdpi.com/article/10.3390/molecules29225286/s1. Table S1: Sedative effect scored of LPM682000012; Table S2: Sedative effect scored of Ganaxolone.
