# Supplementary material for: LPM682000012, a Synthetic Neuroactive Steroid That Ameliorates Epileptic Seizures by Downregulating the Serpina3n/NF-κB Signaling Pathway
Source: Molecules. 2024 Nov 8;29(22):5286. doi: 10.3390/molecules29225286 (PMC11596644; doi:10.3390/molecules29225286)
Supplement: Supplementary file 1 [file molecules-29-05286-s001.zip › molecules-3222450-supplementary.pdf]

Table S1. Sedative effect scored of LPM682000012.

Dose （mg/kg）	Rat#	After drug administration					
		0.5h	1h	1.5h	2h	2.5h	3h
40	1	1	2	2	2	2	death
	2	1	2	2	2	2	2
	3	1	2	2	2	2	2
35	1	1	2	2	2	3	death
	2	1	1	2	2	2	2
	3	1	1	2	2	2	2
30	1	1	1	1	1	1	1
	2	1	1	1	2	1	1
	3	1	1	1	1	2	2
28	1	1	1	1	1	1	1
	2	1	1	1	1	2	1
	3	1	1	1	1	1	1

Table S2. Sedative effect scored of Ganaxolone.

Dose (mg/kg)	Rat#	After drug administration					
		0.5h	1h	1.5h	2h	2.5h	3h
80	1	3	3	4	4	death	
	2	3	4	4	death		
	3	2	2	2	3	3	3
64	1	2	3	3	4	4	death
	2	1	2	2	2	death	
	3	1	2	2	2	2	2
50	1	2	4	4	4	4	death
	2	1	1	1	2	2	1
	3	1	1	1	1	2	2
40	1	1	1	2	3	2	1
	2	1	2	3	3	2	2
	3	1	1	2	3	3	2
